# Investigation of Salivary RANKL and OPG Levels in Periodontitis Patients at Hospital Universiti Sains Malaysia

**DOI:** 10.1055/s-0041-1731930

**Published:** 2021-09-27

**Authors:** Saba Asif, Basaruddin Ahmad, Syed Ameer Hamza, Haslina Taib, Nur Karyatee Kassim, Siti Lailatul Akmar Zainuddin

**Affiliations:** 1 School of Dental Sciences, Health Campus, Universiti Sains Malaysia, Kelantan, Malaysia; 2 Department of Oral Medicine, Dental Hospital, The University of Faisalabad, Pakistan

**Keywords:** periodontitis, receptor activator of NF-ĸB ligand, osteoprotegerin, Saliva

## Abstract

**Objective** This study was aimed to determine the levels of salivary receptor activator of NF-κB ligand (RANKL) and osteoprotegerin (OPG) and its association with periodontal status among periodontitis patients.

**Materials and Methods** A cross-sectional study was designed and performed at the Dental Clinic, Hospital Universiti Sains Malaysia (HUSM). Random sampling was employed to identify 88 participants into three groups: 30 mild periodontitis, 30 moderate to severe periodontitis, and 28 healthy (nonperiodontitis) patients. Periodontal parameters: periodontal pocket depth (PPD), clinical attachment level (CAL), plaque score (PS), and gingival bleeding index (GBI) were recorded. In total, 4 mL of unstimulated whole saliva was collected to determine the levels of salivary RANKL and OPG proteins by using ELISA technique. Data were analyzed by using SPSS software version 24.0.

**Results** Mean values for PPD (5.3 ± 0.5) and CAL (5.6 ± 0.5) were observed higher for moderate to severe periodontitis as compared with values (4.4 ± 0.2) (4.5 ± 0.2) in mild periodontitis patients. The mean salivary RANKL and OPG was 0.23 ± 0.07 ng/mL and 1.78 ± 0.70 ng/mL respectively in moderate to severe periodontitis. Only salivary RANKL levels were significantly and positively correlated with all the clinical periodontal parameters.

**Conclusion** The levels of salivary RANKL were higher as opposed to lower OPG levels in periodontitis patients in contrast to healthy (nonperiodontitis) patients. RANKL levels were significantly associated with the periodontal parameters. Therefore, we can conclude that RANKL can potentially aid as an adjunctive diagnostic protein in evaluating periodontal disease.

## Introduction

Periodontal infection is a slow progressive condition responsible for both inflammation as well as undermining of tooth supporting framework as a result of diverse polymicrobial infection and immune responses in susceptible individuals. 1 Presence of pathogenic anaerobic microorganisms, 2 plaque biofilm, and host immune reactions are considered as main or primary etiological components for this disease. 3 In this case, the regulation of disease pathogenesis comprises interactions between immune reactions from the host along with body response to the bacterial plaque. 4 Several prominent mediators of inflammation including interleukin (IL)-1 β, IL-6, prostaglandin E2, along with tumor necrosis factor (TNF)-α, are released within the body, and these substances activate lytic enzymes and cause disintegration of the alveolar bone. 5 6 7

NF-kB ligand (RANKL) belonged to the renowned family of TNF receptor, and its activation leads to bone resorption and osteoclast precursor cell differentiation. 8 RANKL is produced through various cells such as osteoblasts or stromal cells, 9 fibroblasts, 10 11 and activated B and T cells. 12 The surface of osteoclast and preosteoclast cells is covered with RANK receptors that are activated by RANKL. 13 Binding of RANK with RANKL uplifts the differentiation process for osteoclastic progenitor cells as well as enhances the activity of mature osteoclastic cells. 14

Osteoprotegerin (OPG) also belongs from TNF receptor family with structural resemblance to RANK. 15 OPGs are commonly produced via human periodontal ligament (PDL) cells, epithelial cells, and gingival fibroblasts 16 and further regulated through inflammatory cytokines. 17 OPG functions as antagonist against RANK specific receptor, competitively binding itself onto the surface of RANK molecules. Thus, not only it halts the coupling of RANKL receptors with RANK, but also impedes the differentiation process for both osteoclasts and osteoclastic activity within the cells. 18

Bone remodeling (both bone formation and resorption) is a process which is controlled by RANKL, RANK and OPG system since they are considered primary key factor for bone metabolism. 19 RANK, RANKL, and OPG have been studied for their contribution to the pathological and physiological aspects concerning bone resorption; this has also been researched and discussed previously. 20 21 Considering the specified reasons, we believe that RANK, OPG, and RANKL have a decisive role in regulating the degeneration of periodontal hard and soft supporting tissue.

Current studies exploring the breakdown of alveolar bone leading to periodontal tissue degeneration demonstrate limitations. Clinical studies evaluating RANKL, OPG levels in gingival crevicular fluid (GCF) interpreted that high RANKL, and low OPG protein levels participate in the underlying process of periodontal tissue breakdown. 22 23 24 Interaction of RANKL, OPG proteins with bacterial by products, and inflammatory mediators during periodontitis disease triggers and controls the degenerative process of periodontal tissue breakdown. Till today, none of the previous studies have provided any standard level of RANKL and OPG proteins within whole saliva involved in periodontitis; therefore, this is the first study that was conducted in Malaysian population to evaluate the association of RANKL, OPG proteins using clinical periodontal parameters in both periodontitis and healthy (nonperiodontitis) groups. This study also intended to determine the precise concentrations of both RANKL and OPG proteins in whole saliva from healthy patients and patients with periodontitis. Additionally, we also observed the correlation of these proteins with clinical periodontal parameters in both study groups.

## Materials and Methods

A cross-sectional study was performed at the School of Dental Sciences, Hospital Universiti Sains Malaysia (HUSM). A total of 88 patients from outpatient and periodontics clinic were randomly recruited via probability sampling method and were assigned into three groups: healthy (nonperiodontitis), mild periodontitis, and moderate to severe periodontitis. Patients recruited for this study were already diagnosed with periodontitis. The sample size for salivary RANKL; OPG evaluation in periodontitis patients was calculated by using plaque score (PS) calculator. Calculated sample size for RANKL and OPG protein turned out 27 for each of the three groups using a reference study values. 25 Keeping in account 10% drop out rate of patients, the sample size was round off to 30. Patients constituting mild periodontitis and moderate to severe periodontitis were diagnosed based on case definition by Eke et al. 26 Exclusion criteria consisted of pregnant females, patients having less than 20 permanent teeth, suffering from any systemic disease, on medication, and an antibiotic therapy during the last 3 months. Study approval was obtained and granted by the Human Research Ethics Committee of USM. All patients were made aware of the study, and informed written consent was taken from all patients who wanted to participate.

All the patients were instructed to follow an overnight fast followed by saliva sampling in the morning; during whole procedure, no food or drink was allowed (except water). Prior to clinical measurements, whole saliva samples were obtained and collected via spit out method into the polypropylene tubes. This procedure took about a maximum of 5 minutes for each patient, and both time and volume of collected sample were noted accordingly. 27 28 Samples incorporating any blood particles were discarded and collected again. A domestic refrigerator was used initially to store the samples within 30 minutes of sampling procedure later on samples were freeze at or below −20°C until the day of assay analysis. 28

All teeth (except that of all existing third molars) were inspected for all six sites (mesiobuccal, midbuccal, distobuccal, mesiolingual,, midlingual, and distolingual) by one examiner. Periodontal health status was measured by using Michigan O periodontal probe, and it comprised of plaque index, periodontal probing depth (PPD), gingival bleeding index (GBI), clinical attachment level (CAL). Intra-examiner agreement using intra class correlation coefficient test was used, and the values for correlation coefficients for PPD and CAL came out to be 0.96 and 0.88, respectively.

A clean microcap tube containing 500 mL of individual stored saliva sample was initially clarified by carrying out centrifugation at 10,000 g for approximately 5 minutes; after which the supernatant was relocated into clean microcap tubes; and then an enzyme linked immunosorbent assay (ELISA) was performed. Commercially available salivary RANKL and OPG ELISA kits from Elabscience (USA) were employed to determine the concentrations of both proteins in the saliva samples corresponding to the manufacturers’ manual. Resultant values for salivary RANKL and OPG analysis were represented as ng/mL for concentrations. Standards were incorporated on all rounds. Moreover, all the data along with results are asserted within the given range of the assays.

Statistical evaluation was executed by using ANOVA test to obtain mean concentrations of OPG and RANKL proteins. Pearson’s correlation coefficient analysis was applied to explore the relationship between levels of RANKL and OPG in saliva along with the clinical periodontal parameters. A *p* -value lower than 0.05 was considered statistically significant for this study. This research was conducted by using SPSS software for Windows version 24.0, SPSS Inc., Chicago, Illinois, United States.

## Results

Overall 90 patients participated in the study. However, data of two patients were removed from analysis being incomplete, and data of 88 patients were analyzed. Table 1 provides an insight into the demographic aspects of the participants who were recruited for this study. It was observed that there was an evident and a significant difference for gender between groups. The periodontitis group was composed mostly of female participants and Malay ethnicity.

**Table 1:** Demographic characteristics of study patients in healthy, mild and moderate to severe periodontitis groups (n = 88)

Group	Variables		
	**Age (y)** **(mean ± SD)**	**Gender** **female/male,** *n* **(%)**	**Race** **Malay/Non-Malay** *n* **(%)**
Healthy ( *n* = 28)	30.3 (10.2)	15 (53.5%) 13 (46.5%)	19 (67.8%) 9 (32.2%)
Mild periodontitis ( *n* = 30)	39.1 (10.2)	16 (53.4%) 14 (46.6%)	21 (70%) 9 (30%)
Moderate-to-severe periodontitis ( *n* = 30)	46.0 (10.3)	21 (70%) 9 (30%)	21 (70%) 9 (30%)
*p-* Value ^d^	<0.001 ^a,b,c^	<0.001	<0.001
Abbreviation: SD, standard deviation. ^a^ Post hoc test was significant for age between healthy and mild periodontitis group. ^b^ Post hoc test was significant for age between healthy and severe periodontitis group. ^c^ Post hoc test was significant for age between mild and severe periodontitis group. ^d^*p* < 0.05 is significant.			

In Table 2 , higher mean values for PPD and CAL were observed for patients with severe periodontitis in contrast to mild periodontitis patients. A statistically significant difference was evident among the three study groups in terms of gingival inflammation and mean PS. Moreover, in contrast to other groups in this study, more gingival inflammation and higher mean PSs were observed in the severe periodontitis group.

**Table 2:** Periodontal clinical parameters in healthy, mild and moderate to severe periodontitis groups (n = 88)

Groups	Variables			
	**Mean bleeding on probing (%)**	**Mean plaque scores (%)**	**Mean probing pocket depth (mm)**	**Mean clinical attachment level (mm)**
Healthy (28) Mean (SD)	11.86 ± 5.42	20.86 ± 5.97	00	00
Mild periodontitis (30) Mean (SD)	51.39 ± 17.07	46.34 ± 17.57	4.4 ± 0.2	4.5 ± 0.2
Moderate-to-severe periodontitis (30) Mean (SD)	62.96 ± 17.24	63.21 ± 17.24	5.3 ± 0.5	5.6 ± 0.5
*p-* Value ^d^	<0.001 ^a,b,c^	<0.001 ^a,b,c^	<0.001 ^c^	<0.001 ^c^
Abbreviation: SD, standard deviation. ^a^ Post hoc test was significant between healthy and mild P group. ^b^ Post hoc test was significant between healthy and moderate to severe P group. ^c^ Post hoc test was significant between mild and moderate to severe P group. ^d^*p* < 0.05 is significant.				

Table 3 presents the quantitative determination results for the salivary concentrations of salivary OPG and RANKL. A higher RANKL and OPG levels in severe periodontitis group in contrast with healthy (nonperiodontitis) controls was worth noting in this study.

**Table 3:** Mean values for salivary RANKL and OPG proteins in healthy, mild periodontitis and moderate to severe periodontitis groups

Variable	Salivary RANKL level (ng/mL) Mean (SD) *n* (67)	Salivary OPG level (ng/mL) Mean (SD) *n* (67)	*p-* Value
Healthy	0.08 (0.02)	1.38 (0.72)	<0.001
Mild periodontitis	0.20 (0.11)	1.19 (0.54)	<0.001
Moderate-to-severe periodontitis	0.23 (0.07)	1.78 (0.70)	<0.001
Abbreviations: OPG, osteoprotegerin; RANKL, receptor activator of NF-κB ligand; SD, standard deviation.			

Intergroup differences were analyzed via Dunnett’s formula which are shown in Table 4 . The mean concentration difference for salivary RANKL was observed to be significant between healthy (nonperiodontitis) to mild periodontitis and healthy (nonperiodontitis) to severe periodontitis group, and a nonsignificant finding for mild periodontitis to severe periodontitis groups protein. However, for OPG, the intergroup differences were observed to be nonsignificant for all the three study groups.

**Table 4:** Association of clinical periodontal parameters with salivary RANKL and OPG protein levels in periodontitis patients

	PPD	*p-* Value	CAL	*p-* Value	PS	*p-* Value	GBI	*p-* Value
Salivary RANKL ( *n* = 67)	0.559 ^a^	<0.001	0.554 ^a^	<0.001	0.463 ^a^	<0.001	0.537 ^a^	<0.001
Salivary OPG ( *n* = 67)	0.112	0.367	0.077	0.534	0.121	0.330	0.091	0.466
Abbreviations: CAL, clinical attachment level; GBI, gingival bleeding index; OPG, osteoprotegerin; PPD, periodontal pocket depth; PS, plaque score; RANKL, receptor activator of NF-κB ligand. ^a^ Correlation is significant at the 0.01 level (two-tailed).								

Pearson’s coefficient correlation analysis was performed to assess the association between RANKL and OPG concentrations to periodontal parameters in patients with periodontitis. However, only RANKL salivary concentrations were noted for showing a significant, positive correlation with clinical periodontal parameters, and their variables in this study as depicted in Table 5 .

**Table 5:** Multiple comparison of salivary RANKL and OPG proteins by using Dunnett’s post hoc test

	Groups	Mean difference	Standard error	95% confidence interval	*p-* Value ^a^
Salivary RANKL	Healthy vs. mild Healthy vs. moderate to severe Mild vs. moderate to severe	0.12 0.15 0.03	0.25 0.16 0.02	0.05–0.18 0.10–0.19 −0.42 to 0.10	<0.001 <0.001 0.6
Salivary OPG	Healthy vs. mild Healthy vs. moderate to severe Mild vs. moderate to severe	0.18 0.40 0.59	0195 0.216 0.185	−0.30 to 0.67 −0.13 to 0.94 0.12–1.05	0.7 0.2 0.009
Abbreviations: OPG, osteoprotegerin; RANKL, receptor activator of NF-κB ligand. ^a^*p* < 0.05 is significant.					

## Discussion

Scientists have spent a long period of time attempting to establish a predictable molecular diagnostic marker of periodontal tissue destruction that can be utilized in clinical settings and possess high specificity and sensitivity. 29 Several mediators that are responsible for alveolar bone remodeling experience constant washing into the saliva via GCF. Moreover, unstimulated whole saliva is seen as a dependable substitute to GCF sampling of isolated or specific sites due to the fact that it offers a more enhanced insight for the specific screening of analysis and proteins that are believed to induce the remodeling of bones within the periodontal microenvironment. 30 31

Considering that an escalated salivary RANKL to OPG ratio plays a central part in the pathogenesis of periodontitis infection, 32 33 34 a complete-mouth inspection was utilized to assess and evaluate if the salivary levels of RANKL and OPG proteins are correlated to the clinical outcomes of periodontitis. Thus, all study patients were sorted into three groups; those who do not have periodontitis disease or the group containing healthy patients (nonperiodontitis), patients with mild periodontitis group, and the moderate-to-severe periodontitis group. This study has shown that high RANKL and low OPG protein levels were found significant in patients suffering from severe periodontitis in comparison to nonperiodontitis patients (healthy group). Raised RANKL and OPG levels are responsible for net bone loss and degradation in advanced periodontal lesion. Also, the clinical periodontal parameters (CAL, BOP, PS, and GBI) were more profound in severe periodontitis patients with high RANKL levels when compared with other groups of the study.

It appears that the RANKL and OPG protein levels observed for the saliva samples of both periodontal disease patients and those of healthy (nonperiodontitis) patients corresponded well with the levels detected in GCF samples. However, Frodge et al conducted a study that demonstrated that lower amounts of salivary RANKL was identified in majority of the salivary samples in their study. 35 In part, one can attribute this discrepancy to the sensitivity of the immunoassay, variations in the sampling method, and sample size.

A statistically significant difference in terms of age, gender, race, PD, and CAL counts were observed in terms of clinical as well as demographic aspects of study participants in current study. Likewise, despite it become arduous to compare the outcomes of this study in contrast to other investigations owing to the variations in exploratory technique along with mathematical interpretation of the data.

The results of this study indicated that high RANKL levels were found in patients with moderate to severe periodontal disease in comparison with patients exhibiting mild periodontitis disease and healthy (nonperiodontitis) patients. In a similar manner, there were higher OPG proteins in the severe periodontitis group in contrast to the healthy (nonperiodontitis) group. The data of present study are consistent with all the previously available studies, which collectively indicate that RANKL is increased. However, OPG is decreased in periodontitis compared with healthy (nonperiodontitis) patients. 36 37 38 However, no consensus or guideline exists in terms of the correlation between the RANKL and/or OPG concentrations/or values and the disease’s clinical outcome in terms of CAL, PD, severity, and extent of periodontal degradation. Few earlier investigators including Mogi et al and Lu et al 32 38 were unable to report significant correlations between GCF concentrations of RANKL and/or OPG and clinical measurements. Furthermore, the results of the current investigation agree with a previous report by Bostanci et al, which identified significant positive interrelationship for both salivary RANKL and RANKL/OPG ratio and significant negative correlations for OPG in terms of periodontal clinical status. 22

Despite the previously mentioned observations, both the smoking habit and the age of the patients satisfied the criteria for being classified as confounders, and therefore, it could have a significant influence on data interpretation and the results of the RANKL and OPG. The significant interrelationship between the clinical parameters and age, along with lower OPG values and higher RANKL values detected among the older patients, could also represent periodontal damage’s aging-cumulative characteristics. These results coincide with previous studies, which exhibited significant age-dependent variations observed for RANKL and OPG levels, which potentially suggests that aging promotes the development of the osteoclast progenitor cells, significantly boosts the osteoblastic cell-influenced osteoclast formation and developmental process, changing the linkage and role between osteoclasts and osteoblasts in cancellous bone. 39 40 41

For the prospective studies, we suggest that samples collected from different areas of Malaysia could provide a more generalized description of the prevalence of RANKL and OPG protein levels in periodontitis patients. Additionally, targeting patients diagnosed with only severe periodontitis disease would give a more detailed description of the influence of inflammatory pathway on the levels of salivary RANKL and OPG proteins. A longitudinal designed study would provide a better result for salivary RANKL and OPG in periodontitis patients, specially where postperiodontal therapy changes within the saliva could have been evaluated further as well.

Limitations observed for this study include:

- The study groups recruited patients from one particular state in Malaysia (Kelantan), whereas a diverse range of patients from different states might give a better representation of Malaysian population as a whole.
- The patients only consisted in majority of one type of race, that is, Malay and frequency of Chinese population was low. Representation from more Chinese and other races would give out a diverse representation in the results.
- Larger sample size would have provided a greater depth into the current hypothesis and results. The periodontitis patients in this study belonged to the mild and moderate to severe subcategories. Selecting only the patients with severe periodontitis might give a clearer picture of the level of inflammatory cytokines for diagnostic purposes.
- The diagnostic medium used was saliva, as opposed to plasma serum or GCF which, theoretically, are said to be having higher levels of inflammatory cytokines including RANKL and OPG.

## Conclusion

The present study shows that salivary concentrations for RANKL, OPG proteins along with their ratio do not just benefit as an indicator of the extent and quantity of alveolar bone breakdown and PDL. Instead, there are numerous different confounding factors that could interact and influence the result of this disease, such as aging and different other habits of the susceptible host that may promote tissue destruction and the bone resorption in periodontitis as well.
